# Challenging Cancer (2^nd^ Edn)

**DOI:** 10.1038/sj.bjc.6600672

**Published:** 2003-01-28

**Authors:** A Leary

**Affiliations:** 1 University College, London, UK

‘Challenging Cancer’ is an unusual book. It has a degree of frankness about it, which is refreshing. It is written by an oncologist and a psychotherapist, who give honest accounts of their own experience. I remember reading the first edition of this book as a student. Having made the decision that oncology was for me, I read as much as I could on the subject. This largely consisted of various textbooks until I read ‘Challenging Cancer’. This book broadened my reading and also my understanding of cancer from a patient's perspective. If memory serves, this second edition retains the same excellent format of the first.

‘Challenging Cancer’ is divided into four sections, and although the linking theme is cancer, in essence this book could be four small separate pieces. This gives the content of the book a manageability, particularly for those who have never read a book where cancer is the topic, for example, most of the patient population at which this book is clearly aimed.

The first section details a series of weekend seminars facilitated by a multidisciplinary group that included the authors. The seminars seem to have enabled patients to tell their stories and give perspective at an often confusing time. The second section takes the form of advice on self-help, and perhaps more so a degree of self-empowerment. The third section develops this theme, examining concepts such as crisis and support. This is perhaps the most frank part of the book, as this section uses stories from the experience of the authors to illustrate the intangibles, which from clinical experience we know are important to patients, such as meaning and hope.

The last section integrates well with the rest of the book even though it could stand alone from a historical perspective. It is the story of Vicky Clement Jones, the founder of BACUP. Again the honesty of the authors shows through, particularly the relationship Maurice Slevin shared with Vicky Clement Jones and the breadth of their interactions. The most poignant part, however, is the story Vicky Clement Jones tells about herself, not only about ‘having cancer’ but also her background and why she went into medicine.

‘Challenging Cancer’ is a very emotive book, relying less on factual information about cancer and focussing more on the everyday challenges that people with cancer and the significant people around them face in order to cope with everyday life.

I have no doubt that many patients would find reading ‘Challenging Cancer’ helpful, particularly at milestones, as it is candid and yet tempered with optimism. The only criticism I have of this book is that some of the language used assumes some background knowledge of areas such as psychotherapy. This may make the book less accessible to some groups of patients.

I think this book would also have an impact on anyone embarking on a career in oncology because of its unique and honest approach. ‘Challenging Cancer’ offers insight into a world that is difficult for even the most experienced practitioners in oncology to understand fully. It is a book that is willing to tackle subjects in cancer care that are both challenging and time consuming in everyday practice.

